# Genome Sequence of *Rickettsia gravesii*, Isolated from Western Australian Ticks

**DOI:** 10.1128/genomeA.00975-13

**Published:** 2013-11-27

**Authors:** Erwin Sentausa, Mohammad Yazid Abdad, Catherine Robert, John Stenos, Didier Raoult, Pierre-Edouard Fournier

**Affiliations:** Aix-Marseille Université, URMITE, UM63, CNRS 7278, IRD 198, Inserm U1095, Marseille, Francea; Australian Rickettsial Reference Laboratory, Geelong, Victoria, Australiab

## Abstract

*Rickettsia gravesii* is a new *Rickettsia* species closely related to the human pathogen *Rickettsia massiliae*. Here, we describe the genome sequence of *R. gravesii* strain BWI-1, isolated from *Amblyomma triguttatum triguttatum* ticks collected from humans on Barrow Island, Western Australia.

## GENOME ANNOUNCEMENT

Rickettsiae are obligate intracellular alphaproteobacteria and are the etiological agents of several arthropod-borne diseases in humans. *Rickettsia gravesii* is a novel species isolated from *Amblyomma triguttatum triguttatum* ticks removed from humans on Barrow Island, Western Australia, after there was anecdotal evidence of a disease possibly of rickettsial origin in the region (1). It was also found in other tick species, such as *Amblyomma limbatum* (2); its distribution so far is recognized to coincide with that of *A. triguttatum triguttatum* (3), and it was found to be highly prevalent in members of the latter tick species collected from feral pigs in the southern part of Western Australia (4). Although its pathogenic potential is currently unknown, *R. gravesii* is closely related to the spotted-fever group species *Rickettsia massiliae* (1), which is pathogenic to humans and prevalent in Europe and Africa (5, 6). Here, we describe the genome sequence of *R. gravesii* strain BWI-1^T^.

*R. gravesii* (deposited in the Collection de Souches de l’Unite des Rickettsies [CSUR] under reference R172) was grown in XTC and L929 cells, and its genomic DNA was extracted using a phenol-chloroform protocol. Sequencing was performed using the MiSeq platform (Illumina, San Diego, CA) with a 2 × 250-bp paired-end run after library preparation with the Nextera XT sample preparation kit (Illumina). *De novo* genome assembly was done using the CLC Genomics Workbench 4.9 (CLC bio, Aarhus, Denmark). The resulting contigs were reordered in Mauve 2.3.1 (7) using the genome sequence from *R. massiliae* strain MTU5 (GenBank accession no. CP000683) (8) as a reference. Open reading frame (ORF) prediction and gene annotation were carried out using RAST 4.0 (9). rRNAs, tRNAs, and other RNAs were identified using BLASTn (10), tRNAscan-SE 1.21 (11), and RNAmmer 1.2 (12), respectively. The orthologous genes between *R. gravesii* and *R. massiliae* MTU5 were identified using OrthoMCL (13), with a BLASTp *E* value cutoff of 1 × 10^−5^ and the default Markov cluster algorithm (MCL) inflation parameter of 1.5.

The draft genome sequence of *R. gravesii* BWI-1^T^ is made up of 28 chromosomal contigs exhibiting an average length and coverage of 47,415 bp and 185×, respectively, arranged in a single scaffold, for a chromosome size of 1,327,625 bp (G+C content, 32.2%). We also detected a 19,874-bp plasmid (pRgr) with a G+C content of 31.8% and 91% sequence identity (36% coverage; *E* value, 0.0) to *Rickettsia monacensis* strain IrR/Munich plasmid pRM (accession no. EF564599). The chromosome contains 1,675 protein-encoding genes and, like other *Rickettsia* species, 3 noncontiguous rRNAs (5S, 16S, and 23S rRNA), 33 tRNAs, and 3 other RNAs. In addition, the pRgr plasmid contains 24 protein-encoding genes, including a split *sca12* gene and a proline-betaine transporter gene, but no RNAs.

Compared to that of *R. massiliae* MTU5, the *R. gravesii* chromosome exhibits a high level of synteny with the exception of four inversions of 37,978 bp, 4,791 bp, 2,782 bp, and 1,339 bp. Moreover, several genes are lacking in the *R. gravesii* genome, including *paaJ* (acetyl-coenzyme A [CoA] acetyltransferase), *def*3 (polypeptide deformylase), and genes for several transposases and inactivated derivatives.

### Nucleotide sequence accession number.

This whole-genome shotgun project has been deposited at DDBJ/EMBL/GenBank under the accession no. AWXL00000000.
